# The comparison of depression and anxiety between fertile and infertile couples: A meta-analysis study

**DOI:** 10.18502/ijrm.v17i3.4514

**Published:** 2019-05-29

**Authors:** Hossein Fallahzadeh, Hasan Zareei Mahmood Abadi, Mahdieh Momayyezi, Hakimeh Malaki Moghadam, Naeimeh Keyghobadi

**Affiliations:** ^1^ Research Center of Prevention and Epidemiology of Non-Communicable Disease, School of Public health, Shahid Sadoughi University of Medical Sciences, Yazd, Iran.; ^2^ Department of Psychology, Faculty of Humanities, Yazd University, Yazd, Iran.; ^3^ Department of Statistics and Epidemiology, School of Public health, Shahid Sadoughi University of Medical Sciences, Yazd, Iran.

**Keywords:** *Depression*, * Anxiety*, * Infertility*, * Meta-analysis.*

## Abstract

**Background:**

Depression and anxiety are the most common reaction in infertile couples. Several studies have been conducted to examine the psychiatric disorders among infertile and fertile couples.

**Objective:**

This meta-analysis was conducted to compare the depression and anxiety in fertile and infertile couples in various studies.

**Materials and Methods:**

The authors searched articles published in multiple databases including World Health Organization, PubMed, Cochrane Library, Scopus, Science Direct, Medline EMBASE and Persian databases including Scientific Information Database (SID) and IranMedx between 2005 and 2017. The main keywords used for searching the databases were: depression, anxiety, infertility, and fertility. Statistical analyses were performed using Comprehensive Meta-Analysis/2.0 software.

**Results:**

The authors found 42 related articles after searching the databases. 11 articles entered the meta-analysis after considering the inclusion and exclusion criteria. Finally, eight articles were chosen for the comparison of depression and anxiety, two published articles for the comparison of depression, and one published article to compare anxiety in fertile and infertile couples. The results of the heterogeneity test showed a significant heterogeneity among all articles that were analyzed in this meta-analysis in the field of depression and anxiety. The results showed that depression (p = 0.0001; Hedges'g = 1.21; 95% CI 0.63–1.78) and anxiety (p = 0.00001; Hedges'g = 0.63; 95% CI 0.54–0.73) were higher in infertile couples than fertile couples and that the possibility of a publication bias does not exist in this study.

**Conclusion:**

The analysis of articles used in this meta-analysis showed that depression and anxiety scores in infertile couples were higher than fertile couples.

## 1. Introduction

Infertility is the inability to become pregnant after 12 months or more of regular unprotected sexual intercourse (1). Infertility is one of the personal and social problems affecting couples' life and family functioning and can expose people to psychological stress or psychiatric disorders (2). Infertility may be considered as one of the most stressful events. Researchers reported that Infertility associated with stressful experience, health problems, lack of self-confidence, feeling of grievance, threat, depression, sin, disappointment, and marital problems (3). According to the World Health Organization, about 33.35% (about 5 million) people in the world are infertile (4). Infertility can play a role in generating stress in the family. The stress due to infertility reduces self-esteem in couples. The collection of these emotions leads to depression and anxiety in them (5). Depression and anxiety are the most common reaction in infertile couples (6).

Anxiety and depression are called as two relatively common mental disorders. The prevalence of depressionn in the general population in Iran has been reported between 2.4 and 37%. (7). The overall prevalence of mental disorders in infertile couples is reported to be between 25% and 60% (8). In a study of 55 infertile women (25 males and 30 females), anxiety and depression were found in infertile couples. The most important factor in this issue is the attitude of individuals toward living control and social acceptance (9). In Behdani and colleagues, 57.1% of infertile women had a degree of depression. Also, depression in 20.9% of them was clinical depression (10). The chance of getting pregnant may increase by treating mental disorders. Reducing the anxiety of couples referring to infertility treatment centers through counseling and supportive psychotherapy are effective (3). The presence of clinical psychologists and psychiatrists in diagnostic and treatment centers is important in helping infertile couples to better adapt to stress due to infertility.

Most studies reported that depression and anxiety symptoms were intensified in infertile women undergoing treatment with negative response of pregnancy testing. Many studies have been done to examine the psychiatric disorders among infertile and fertile couples. Therefore, this meta-analysis was conducted to compare the depression and anxiety in fertile and infertile women in various studies.

## 2. Materials and Methods

This article is a systematic review and meta-analysis that conducted based on the PRISMA (Preferred Reporting Items for Systematic Reviews and Meta-Analyses) Checklist (11).

### Literature and search strategy 

Researchers searched articles in multiple databases including World Health Organization, PubMed, Cochrane Library, Scopus, Science Direct, Medline EMBASE, and Persian databases such as SID and Iran Medx. All studies about the depression and anxiety among fertile and infertile couples published between 2005 and 2014 were collected. The main keywords used for searching the databases were: depression, anxiety, infertility, and fertility.

### Inclusion and exclusion criteria

The inclusion criteria for this meta-analysis were: 1) cohort and case-control studies; 2) Studies that show depression and anxiety in fertile or infertile couples; 3) articles that reported mean and SD for depression and anxiety. Also, the exclusion Criteria included: 1) same studies that published in several journals (researchers included more recent studies into the meta-analysis) and 2) case reports, editorials, review articles, conference papers, meta-analysis studies.

### Data extraction

Data extraction was carried out using two readers independently. Researchers recorded the number of infertile and fertile groups, depression and anxiety ascertainment, subjects, mean of depression and anxiety in infertile and fertile group.

### Quality assessment

The quality of the methodology of studies was evaluated by The Newcastle–Ottawa Quality scale. On this scale, articles with higher quality receive 9 stars (12).

### Ethical consideration

This article has a license from the research ethics committees of Shahid Sadoughi University of Medical Sciences, code IR.SSU.SPH.REC.1394.81.

### Statistics and analysis

We used I${}^{2}$ index and Q test to assess quantitative heterogeneity in meta-analysis (p < 0.1). I² ranges 0–100% and describes the percentage of inconsistency across studies in a meta-analysis. A value of 0% indicates no heterogeneity between the studies.

All of the sample estimate transferred to standardized mean difference effect sizes (d) and unbiased sample estimate standardized mean difference effect sizes (g, also known as Hedges' g like Cohen' d) (13). The Hedges' g of 0.2 identified as a small difference between groups, 0.5 a moderate difference, and 0.8 ≤ a large difference. These values suggest statistical differences measured in terms of effect size; while clinical measures of anxiety and depression are suitable for assessing clinical differences.

In addition, publication bias (p < 0.05) was determined by linear regression Egger's and Begg's rank correlation and funnel plot. Comprehensive Meta-Analysis/2.0 was used for statistical analyses.

## 3. Results

**Figure 1:**
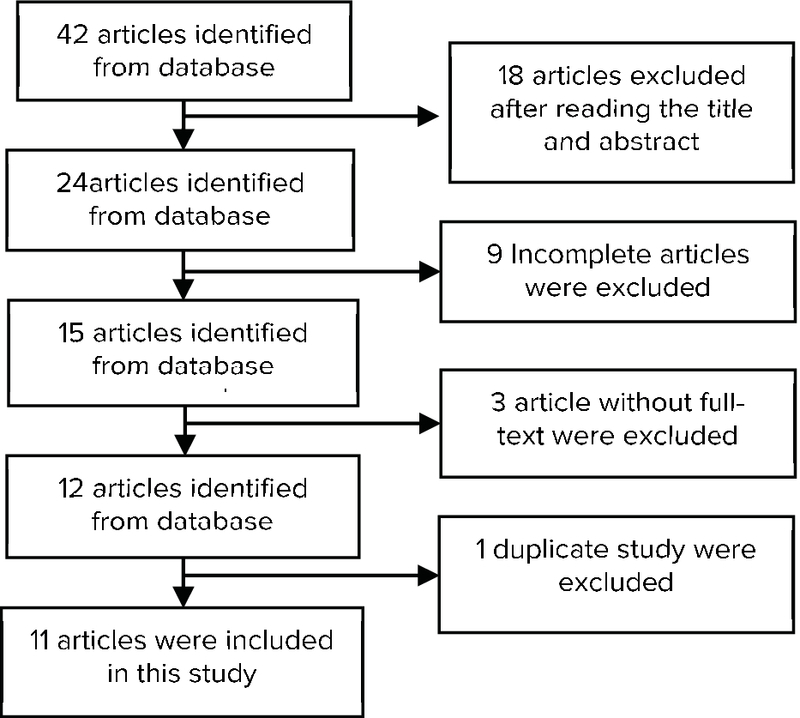
Flow chart of study selection

Considering the title, abstract, and inclusion and exclusion criteria, 42 articles were entered in the meta-analysis. Finally, eight studies were considered for the comparison of depression and anxiety, two studies for the comparison of depression, and one published article to compare anxiety in fertile and infertile couples (Figure 1).

The quality of the methodology of studies was evaluated using the Newcastle–Ottawa Assessment Scale (Tables I and II). The results show that articles entered into this study received 5 to 8 stars, also nine studies have high quality (overall score > 6). Table III shows the main characteristics of the eligible studies. All articles were written in English language except two that were published in Persian language. Also, five of the studies were conducted in Iran; two were from turkey, one from Korea, one from Italy, one article from Nigeria, and one from Poland. Additionally, five of the studies were conducted during 2002–2008 and six of them during 2011–2016.

**Figure 2:**
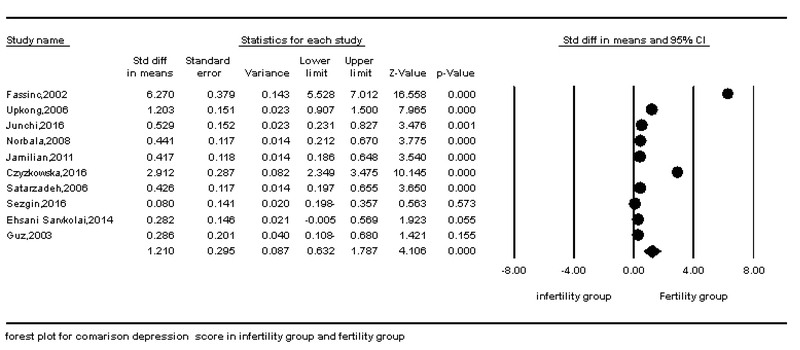
Forest plot of the comparison of depression in fertile and infertile couples

**Figure 3:**
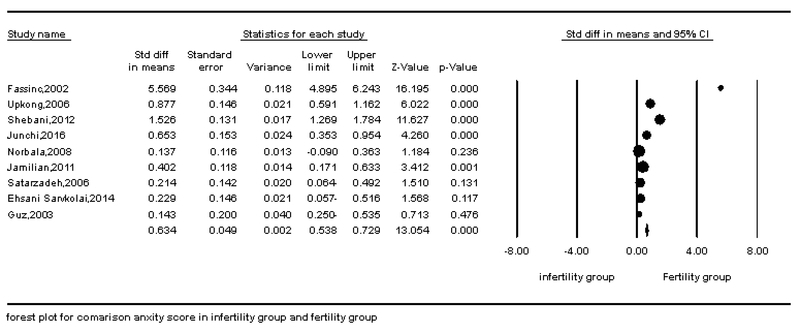
Forest plot of the comparison of anxiety in fertile and infertile couples

**Figure 4:**
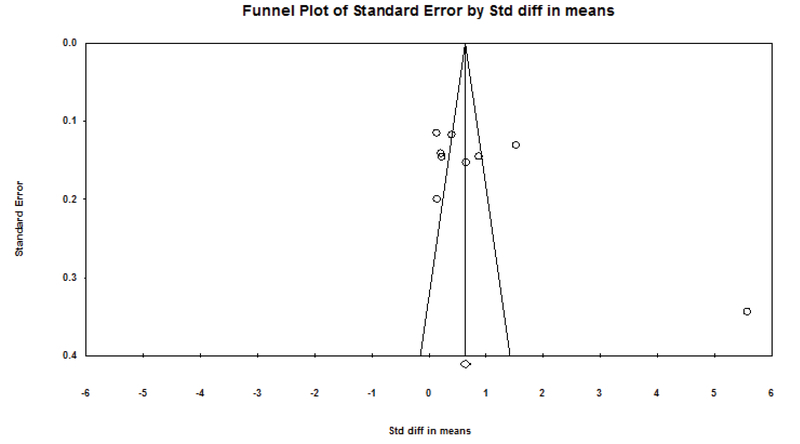
Funnel plot of the comparison of anxiety in fertile and infertile couples

**Figure 5:**
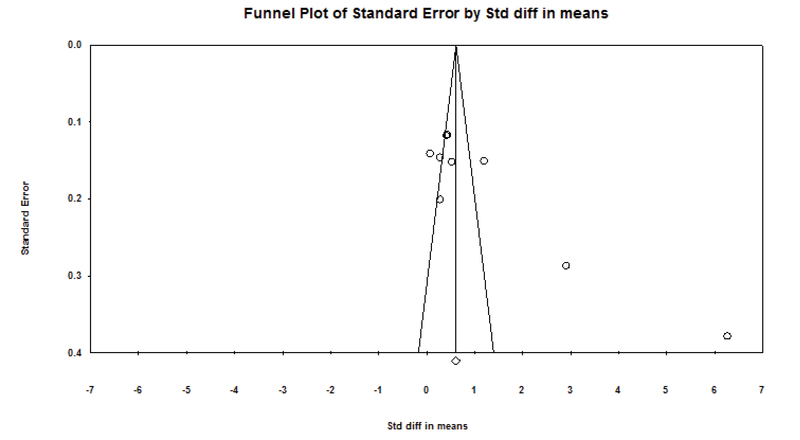
Funnel Plot of the comparison of depression in fertile and infertile couples

According to the survey conducted in this meta-analysis, depression and anxiety in fertile and infertile couples have a statistically significant association in all articles. The results of this study showed that the standard effect size (hedges' g) for the difference in anxiety scores in the infertile couples than the fertile couples was 0.653. This finding showed a moderate difference in anxiety between fertile and infertile couples; so that the anxiety levels were higher in infertile couples than fertile couples. In addition, the standard effect size (hedges' g) for the difference in depression scores in the infertile group than the fertile group was 1.21. This finding showed a large difference in depression between fertile and infertile couples; so that the depression levels were higher in infertile couples than fertile couples. The STD mean obtained by the fixed effects model and random effect and Cochran test results are shown in Table IV. The results of Table IV showed that Cochran's test was significant (p < 0.0001), therefore, researchers used the random effect model for analysis. The results of the heterogeneity test showed a significant heterogeneity among all articles that were analyzed in this meta-analysis in the field of depression (I${}^{2}$ = 97.29, Q-value = 332.35, p = 0.0001). Also, there was a significant heterogeneity among all articles in the field of anxiety that were analyzed in this meta-analysis (I${}^{2}$ = 97.33, Q-value = 299.76, P-value = 0.0001 (Figures 2 and 3)). Begg's test and Egger's test for depression (p = 0.06 and p = 0.0736, respectively) and anxiety (p = 0.21 and p = 0.251, respectively) showed that there is no probable of publication bias in this meta-analysis. Funnel plot is shown in Figures 4 and 5.

**Table 1:** The methodological quality of studies based on Newcastle–Ottawa scale (case-control study)


	**Selection**				**Comparability**	**Outcome**	
	Is the case definition adequate?	Representativeness of the cases	Selection of Controls	Definition of Controls	Comparability of cases and controls on the basis of the design or analysis	Ascertainment of exposure	Same method of ascertainment for cases and controls	Non-response rate	Total
Ehsani Sarvkolai, Iran, 2014 (14)	*	*	- *	*	*	- -	5
Fassino, Italy, 2002 (15)	*	*	- *	*	*	*	- 6
Guz, Turkey, 2003 (16)	*	*	*	*	*	*	*	*	8
Jamilian, Iran, 2011 (17)	*	*	*	*	**	*	*	- 8
Noorbala, Iran, 2008 (3)	*	*	- *	*	*	- -	5
Upkong, Nigeria, 2006 (18)	*	*	*	*	*	*	*	- 7
Satarzadeh, Iran, 2006 (19)	*	*	- *	*	*	*	- 6
Ara-Sheybani, Iran, 2012 (20)	*	*	*	*	*	*	*	*	8
Czyzkowska, Poland, 2016 (21)	*	*	*	- **	*	*	*	8
Sezgin, Turkey, 2016 (22)	*	*	*	*	*	*	*	- 7

**Table 2:** The methodological quality of studies based on Newcastle–Ottawa scale (cohort study)


	**Selection**				**Comparability**		**Outcome**		
	Representativeness of the exposed cohort	Selection of the non-exposed cohort	Ascertainment of exposure	Demonstration that outcome of interest was not present at the start of study	Comparability of cohorts on the basis of the design or analysis	Assessment of outcome	Was follow-up long enough for outcomes to occur	Adequacy of follow-up of cohorts	Total
Chi, Korea, 2016 (23)	*	*	*	- *	*	–	*	6

**Table 3:** Characteristics of the studies correlating the comparison of depression and anxiety in fertile and infertile couples


**Name, country, year (ref.)**	**Number of infertile group**	**Number of fertile group**	**Depression & Anxiety ascertainment**	**Subjects**	**Mean in infertile group**	**Mean in fertile group**
Ehsani Sarvkolai, Iran, 2014 (14)	100	89	Beck Depression inventory	Depression	15	17.9
		ZANK	Anxiety	35.67	37.15
Fassino, Italy, 2002 (15)	85	80	Hamilton depression	Depression	14.15	3.23
		Hamilton Anxiety	Anxiety	14.86	4.31
Guz, Turkey, 2003 (16)	50	50	SCL-90-R	Depression	1	0.8
		Anxiety	0.8	0.7
Jamilian, Iran, 2011 (17)	147	147	General Health Questionnaire-(GHQ)	Depression	5.16	3.57
		Anxiety	7.94	6.35
Chi, Korea, 2016 (23)	141	65	Depression Anxi­ety Stress Scales (DASS)	Depression	13.7	9.4
		Anxiety	10.7	6.6
Noorbala, Iran, 2008 (3)	150	150	SCL-90-R	Depression	1.3	0.98
		Anxiety	1	0.9
Upkong, Nigeria, 2006 (18)	112	96	Hospital Anxiety and	Depression	9.02	2.83
		Depression Scale		
		Beck Depression inventory	Anxiety	6.08	2.51
Satarzadeh, Iran, 2006 (19)	100	100	Beck Depression inventory	Depression	16.19	11.44
Ara-Sheybani, Iran, 2012 (20)	155	145	Infertility Distress Scale (IDS)	Anxiety	59.9	42.85
Czyzkowska, Poland, 2016 (21)	50	50	Beck Depression inventory	Depression	16.64	2.44
Sezgin, Turkey, 2016 (22)	100	100	Hospital Anxiety and Depression Scale (HADS)	Depression	6.6	6.3
		Anxiety	8.2	7.3

**Table 4:** Heterogeneity test results of the studies correlating the study of depression and anxiety in fertile and infertile couples


**Type of model**	Point STD mean*	**P-value**	**Confidence Interval**	**Q-value	**p-value**	**I²**
Depression						
Fixed effect	0.61	0.000	(0.51–0.7)	332.35	0.000	97.29
Random effect	1.21	0.000	(0.63–1.78)	–	–	–
Anxiety						
Fixed effect	0.63	0.000	(0.55–0.78)	299.76	0.000	97.33
Random effect	1.03	0.001	(0.44–0.96)		
Note: *STD: Standardized mean difference; I${}^{2}$: I-square; **Q: the Q value for the heterogeneity Q test for between-subgroup differences.						

## 4. Discussion

Infertility can be *described* as a crisis that is associated with physical, psychological, mental, and social problems. Infertile women are often driven by their husband and society due to their inability to give birth; this has a negative impact on their psychological state. The results of this study showed that the standard effect size (hedges' g) for the difference in anxiety scores in the infertile group than the fertile group was 0.653, and this relationship was statistically significant (p = 0.0001). The standardized mean difference is considered as a noticeable effect size. This value has been positive in the present study; the results showed that the anxiety levels were higher in infertile couples than fertile couples in all the studies reviewed. Of course, the effect size of hedges' g can be converted to the odds ratio index for further interpretation. The result of this conversion was 3.25 for the odds ratio. This showed that infertile women have three times more chance of anxiety than fertile women. These findings are consistent with separate studies conducted by various researchers (15, 17, 18, 20, 23). Therefore, psychological interventions, especially cognitive supportive therapies, may be useful to reduce anxiety in infertile women. The results of this study showed that the standard effect size (hedges' g) for the difference in depression scores in the infertile group than the fertile group was 1.21, and this relationship was statistically significant (p = 0.0001). This value has been positive in the present study; the results showed that depression levels were higher in infertile women than fertile women. The result of the conversion of the effect size to the odds ratio was 8.97. This suggests that the depression in infertile couples is about nine times higher than fertile couples. The analysis of 11 articles used in this meta-analysis showed that depression and anxiety scores in infertile couples were higher than fertile couples. This difference has been shown in many national and international studies. Of course, there are exceptions in this relationship. Sezgin and co-worker did not show a significant difference between depression and anxiety in infertile and fertile couples. In their study, only clinical anxiety in infertile couples was significantly higher than fertile couples (22). People who experience infertility need psychological support to deal with mental problems. Therefore, mental and emotional support must be integrated with clinical support. Upkong in his study showed that age, lack of at least one child, and lack of support from the husband were the predictors of depression and anxiety in infertile women (18). Obstetricians should consider that reducing mental and social problems may lead to increased treatment satisfaction and fertility. If depression and anxiety are chronic, it may have a negative effect on the treatment. Sometimes depression and anxiety turn into isolation and lack of eagerness to participate in society. This is a serious issue and shows the role of social support. The limitations of the present study are as follows (1). The information from subgroups is not mentioned in the results of the articles used in this meta-analysis, so researchers cannot use them in the subgroup analysis (2). Different tools have been used to measure anxiety and depression in the articles.

## 5. Conclusion

The analysis of articles used in this meta-analysis indicated that depression and anxiety scores in infertile couples were higher than fertile couples. Therefore, increasing the awareness of infertile couples by using workshops, providing educational packages, informing through mass media, providing free counseling services, and educating the general public and families about new infertility treatment technologies can help in reducing anxiety, depression, and mental disturbances. Therefore, it is recommended to the family counselors that provide programs for increasing the level of knowledge of couples in this regard.

##  Conflict of Interest 

The authors declare that there is no conflict of interest.

## References

[1] Monga Manoj, Alexandrescu Bogdan, Katz Seth E., Stein Murray, Ganiats Theodore (2004). Impact of infertility on quality of life, marital adjustment, and sexual function. Urology.

[2] Boivin J (2003). A review of psychosocial interventions in infertility. Social Science & Medicine.

[3] Noorbala Ahmad Ali, Ramezanzadeh Fatemeh, Abedinia Nasrin, Naghizadeh Mohammad Mehdi (2008). Psychiatric disorders among infertile and fertile women. Social Psychiatry and Psychiatric Epidemiology.

[4]   4. Younesi SJ, Salagegheh A. Body image in fertile and infertile women. J Reprod Fertil 2001; 2: 14-21..

[5]   5. Peyvandi S, Hosseini SH, Daneshpour MM, Mohammadpour RA, Qolami N. [The prevalence of depression, anxiety and marital satisfaction and related factors in infertile women referred to infertility clinics of Sari city in 2008.] J Mazandaran Univ Med Sci 2011; 20: 26-32. (In Persian).

[6] Schmid Julia, Kirchengast Sylvia, Vytiska-Binstorfer Elisabeth, Huber Johannes (2004). Infertility caused by PCOS—health-related quality of life among Austrian and Moslem immigrant women in Austria. Human Reproduction.

[7]   7. Farzaneh F, Momayyezi M, Lotfi MH. Relationship between quality of sleep and mental health in female students of Shahid Sadoughi University of Medical Sciences (2015). J Fundamentals of Ment Health 2018; 20: 167-171..

[8] Wallach Edward E., Seibel Machelle M., Taymor Melvin L. (1982). Emotional aspects of infertility. Fertility and Sterility.

[9]   9. Nilforooshan P, Ahmadi SA, Abedi MR, Ahmadi SM. [Attitude towards infertility and its relation to depression and anxiety in infertile couples.] J Reprod Fertil 2006; 6: 546-552. (in Persian).

[10]   10. Behdani F, Mousavifar N, Hebrani P, Soltanifar A, Mohamadnejad M. Anxiety and mood disorders in infertile women referred to Montaserie infertility clinic in Mashhad, North-East Iran. Iran J Obstet Gynecol Infertil 2008; 11: 15-23..

[11] Moher D., Liberati A., Tetzlaff J., Altman D. G,   (2009). Preferred reporting items for systematic reviews and meta-analyses: the PRISMA statement. BMJ.

[12] Simou Evangelia, Britton John, Leonardi-Bee Jo (2018). Alcohol and the risk of sleep apnoea: a systematic review and meta-analysis. Sleep Medicine.

[13] Hedges Larry V. (1981). Distribution Theory for Glass's Estimator of Effect size and Related Estimators. Journal of Educational Statistics.

[14]   14. Ehsani Sarvkolai H, Shahidi M, Yaghoubi A. The comparison of depression, anxiety, self-esteem, and life satisfaction between fertilized and infertilized women. Trend Life Sci 2014; 3: 97-101..

[15] Fassino S., Piero A., Boggio S., Piccioni V., Garzaro L. (2002). Anxiety, depression and anger suppression in infertile couples: a controlled study. Human Reproduction.

[16] Guz H., Ozkan A., Sarisoy G., Yanik F., Yanik A., Schuiling G. A. (2003). Psychiatric symptoms in Turkish infertile women. Journal of Psychosomatic Obstetrics & Gynecology.

[17]   17. Jamilian M, Rafiei M, Jamilian HR, Esmkhani A. The comparison of general health between fertile and infertile women of Arak City in 2010. J Arak Uni Med Sci 2012; 14: 27-35..

[18]   18. Upkong D, Orji E. [Mental health of infertile women in Nigeria]. Turk Psikiyatri Derg 2006;17: 259-265..

[19]   19. Satarzadeh N, Bahrami N, Ranjbar Kouchaksaraei F, Ghoujazadeh M. Comparison of sexual satisfactory and depression between sterile and unsterile couples refer to alzahra research and education center, Tabriz. Yafteh 2007; 9: 17-24..

[20]   20. Arab-Sheybani K, Janbozorgi M, Akyuz A. Admissibility investigation and validation of infertility distress scale (IDS) in iranian infertile women. Int J Fertil Steril 2012; 6: 37-44..

[21]   21. Czyzkowska A, Awruk K, Janowski K. Sexual satisfaction and sexual reactivity in infertile women: the contribution of the dyadic functioning and clinical variables. Int J Fertil Steril 2016; 9: 465-476..

[22]   22. Sezgin H, Hocaoglu C, Guvendag-Guven ES. Disability, psychiatric symptoms, and quality of life in infertile women: a cross-sectional study in Turkey. Shanghai Arch Psychiat 2016; 28: 86-94..

[23] Chi Hee-Jun, Park Il-Hae, Sun Hong-Gil, Kim Jae-Won, Lee Kyeong-Ho (2016). Psychological distress and fertility quality of life (FertiQoL) in infertile Korean women: The first validation study of Korean FertiQoL. Clinical and Experimental Reproductive Medicine.

